# A systematic review of determinants of breast cancer risk among women with benign breast disease

**DOI:** 10.1038/s41523-024-00703-w

**Published:** 2025-02-15

**Authors:** Aileen Burke, Jessica O’Driscoll, Mustapha Abubakar, Kathleen E. Bennett, Emma Carmody, Fidelma Flanagan, Gretchen L. Gierach, Maeve Mullooly

**Affiliations:** 1 School of Population Health, RCSI University of Medicine and Health Sciences, Dublin, 2 Ireland; 2 Division of Cancer Epidemiology and Genetics, National Cancer Institute, Bethesda, MD USA; 3 BreastCheck, National Screening Service, Dublin, Ireland

**Keywords:** Risk factors, Cancer epidemiology, Breast cancer, Cancer prevention

## Abstract

Benign breast disease (BBD) is associated with heterogeneous breast cancer risk. Identifying key breast cancer risk factors for this population may inform breast cancer prevention or early detection strategies. We systematically searched literature databases PubMed, Embase, Scopus, Web of Science, and the Cochrane Library to identify studies reporting associations of demographic, lifestyle, reproductive, and radiological factors with risk of breast cancer among women with biopsy-confirmed BBD. 67 studies met eligibility criteria. Variation was observed for study time period, exposure measurement, comparison groups, outcomes, and adjustment for confounders, precluding meta-analysis. The literature suggested positive risk associations for age at biopsy, family history, mammographic breast density, and time since biopsy, and no association for body mass index, alcohol, smoking, age at menarche, and use of hormonal contraceptives. More research is needed to understand risk factor associations among women with BBD, particularly studies that account for heterogeneity within BBD and breast cancer.

## Introduction

Benign breast disease (BBD) represents a heterogeneous group of lesions corresponding to a spectrum of abnormal breast tissue changes^1^. It is typically diagnosed on biopsy following detection of a palpable or mammographic abnormality^1^. Women with BBD are at heterogeneous risk of breast cancer, supporting the hypothesis that these lesions represent non-obligate breast cancer precursors^1^. Risk varies according to the degree of epithelial proliferation and whether atypia is present (i.e. whether some, but not all, features of low-grade carcinoma in situ are observed^1^). Compared to non-proliferative disease, epithelial proliferation without atypia confers an approximately 2-fold increased breast cancer risk (relative risk (RR) 1.76, 95% confidence interval (CI): 1.58–1.95), while epithelial proliferation with atypia confers an approximately 4-fold risk increase (RR 3.93, 95% CI: 3.24–4.76)^2^. Additional evidence suggests that multiple foci of benign lesions further increase risk across both breasts, irrespective of the histology of the lesion^3^. However, while histological classification facilitates clinical management, each subgroup comprises multiple lesions with heterogeneous malignant potential^1,4^.

It is thus important to understand not only the influence of benign lesions on subsequent breast cancer risk, but also the risk in the presence of additional demographic, lifestyle, reproductive, or radiological risk factors for breast cancer. While BBD is included in several risk prediction models^5–7^, they have been shown to have modest ability to predict breast cancer among women with BBD^8–10^. A BBD-specific risk prediction model was developed in the Mayo Clinic BBD cohort^11^ and has been found to have improved discriminatory accuracy among women with BBD compared to the widely-used Gail model and the Breast Cancer Surveillance Consortium model^12^. However, the Mayo Clinic BBD model does not include mammographic breast density (MBD), a strong risk factor for breast cancer^13^. Furthermore, there is known uncertainty in the use of population-level models to predict individual risk^14^.

Much research to date has evaluated the association of breast cancer risk factors with risk of breast cancer, with fewer studies investigating the independent contributions of breast cancer risk factors among women with BBD to breast cancer risk^15–22^. Within this restricted systematic review, we aimed to examine the literature for studies investigating associations of risk factors with risk of breast cancer among women with biopsy-confirmed BBD. Improved awareness of factors that increase breast cancer risk among this population may inform clinical management, tailored prevention and early detection strategies.

## Methods

### Study design and reporting

The Preferred Reporting Items for Systematic Review and Meta-Analysis (PRISMA) guidelines were used to report this review^23^ (Supplementary Table 1). The protocol for this review was registered with the International Prospective Register of Systematic Reviews (PROSPERO^24^). We have termed this a restricted systematic review, following the recommendations of Plüdemann et al.^25^, to indicate that methodology was simplified in the areas of full-text review, data extraction, and risk of bias assessment as they were carried out by a single reviewer, and that the results were summarised narratively.

### Data sources and search strategy

A comprehensive search strategy was developed in consultation with a medical librarian (Supplementary Table 2). Citations were identified by searching PubMed (pubmed.ncbi.nlm.nih.gov), EMBASE (embase.com), Web of Science (webofknowledge.com), Scopus (scopus.com), and the Cochrane Library (cochranelibrary.com). The search dates were from the beginning of the index to November 2022. The reference lists of studies that were included following full-text review were also screened for citations not captured by the database search.

### Study eligibility criteria

Studies were eligible for inclusion if they included populations of women aged 18 years or older who had a diagnosis of BBD confirmed on biopsy, and who were evaluated for risk of breast cancer associated with at least one risk factor. Studies reporting BBD diagnosed by clinical breast exam, fine needle aspiration, imaging, or without explicit reference to biopsy confirmation were not included. No publication date restrictions were applied. Non-English language studies, reviews, conference abstracts, protocols, editorials, letters, case studies, and in vitro and animal studies were excluded.

### Screening and study selection process

Title and abstract screening was carried out by a single reviewer (AB), with a random sample of 50% of the citations independently screened by an additional reviewer (JOD). Any disagreement was resolved by discussion of the article to reach consensus. Full text review was carried out by a single reviewer (AB).

### Data extraction

Data extraction was carried out by a single reviewer (AB). Details regarding study design, setting, analytic population, risk factors, follow-up and outcomes, methodology, and risk estimates were extracted.

### Exposure/risk factors

BBD histological categories were defined as non-proliferative disease (NPD), proliferative disease without atypia (PDWA), and atypical hyperplasia (AH)^1^. Demographic risk factors comprised age at benign biopsy, body mass index (BMI), chemoprevention, family history of breast cancer, previous benign biopsies, and time since biopsy. Lifestyle risk factors comprised alcohol consumption, use of non-steroidal anti-inflammatory agents (NSAIDs), physical activity, and smoking. Reproductive risk factors comprised age at menarche, use and duration of hormonal contraceptives, parity, age at first birth, menopausal status at biopsy, age at menopause, bilateral oophorectomy, and use and duration of menopausal hormone therapy (MHT). Radiological risk factors comprised calcifications and MBD.

### Outcome measures

The outcome measure was the risk estimate for invasive or in situ breast cancer among women with biopsy-confirmed BBD for each risk factor identified. Estimates were extracted for three different comparison groups: comparisons within BBD cohorts; women with BBD compared to women without BBD; and population-based breast cancer incidence rates. Extracted risk estimates included relative risk (RR), odds ratio (OR), hazards ratio (HR), standardised incidence ratio (SIR), and incidence rate ratio (IRR), with the associated 95% confidence interval (CI).

### Quality appraisal and risk of bias assessment

All included studies were appraised for methodological quality and risk of bias using the relevant Joanna Briggs Institute (JBI) checklist based on the study design (cohort or case-control^26^; Supplementary Tables 5 and 6) by a single reviewer (AB). For each item on the checklist, a score of 1 was assigned if the criterion was met. No score was given if a study did not meet the criterion, if it was unclear, or not reported. Studies were classified according to the number of unmet criteria as low risk of bias (0 or 1 unmet criteria), moderate risk of bias (2 or 3 unmet criteria), or high risk of bias (more than 3 unmet criteria).

### Data synthesis strategy

Risk of breast cancer among women with BBD was assessed according to each risk factor for each comparison group (women with BBD, women without BBD or reference population incidence rate). For risk factors identified in four or more studies, findings were summarised narratively. For those evaluated in three studies or fewer, study findings were noted but not summarised; we did not exclude any studies from the review on this basis. We also identified instances where included studies were sub-cohort analyses, such as for specific BBD diagnoses^27–34^, or where risk estimates were derived from different comparisons within the same population. Where risk factor estimates were presented both for the overall cohort and for sub-populations or different comparison groups, we noted this and any concordance or divergence observed. Results were presented according to the comparison population, i.e. comparisons to women with BBD, without BBD or to population-based rates of breast cancer.

## Results

### Search selection

**Fig. 1:**
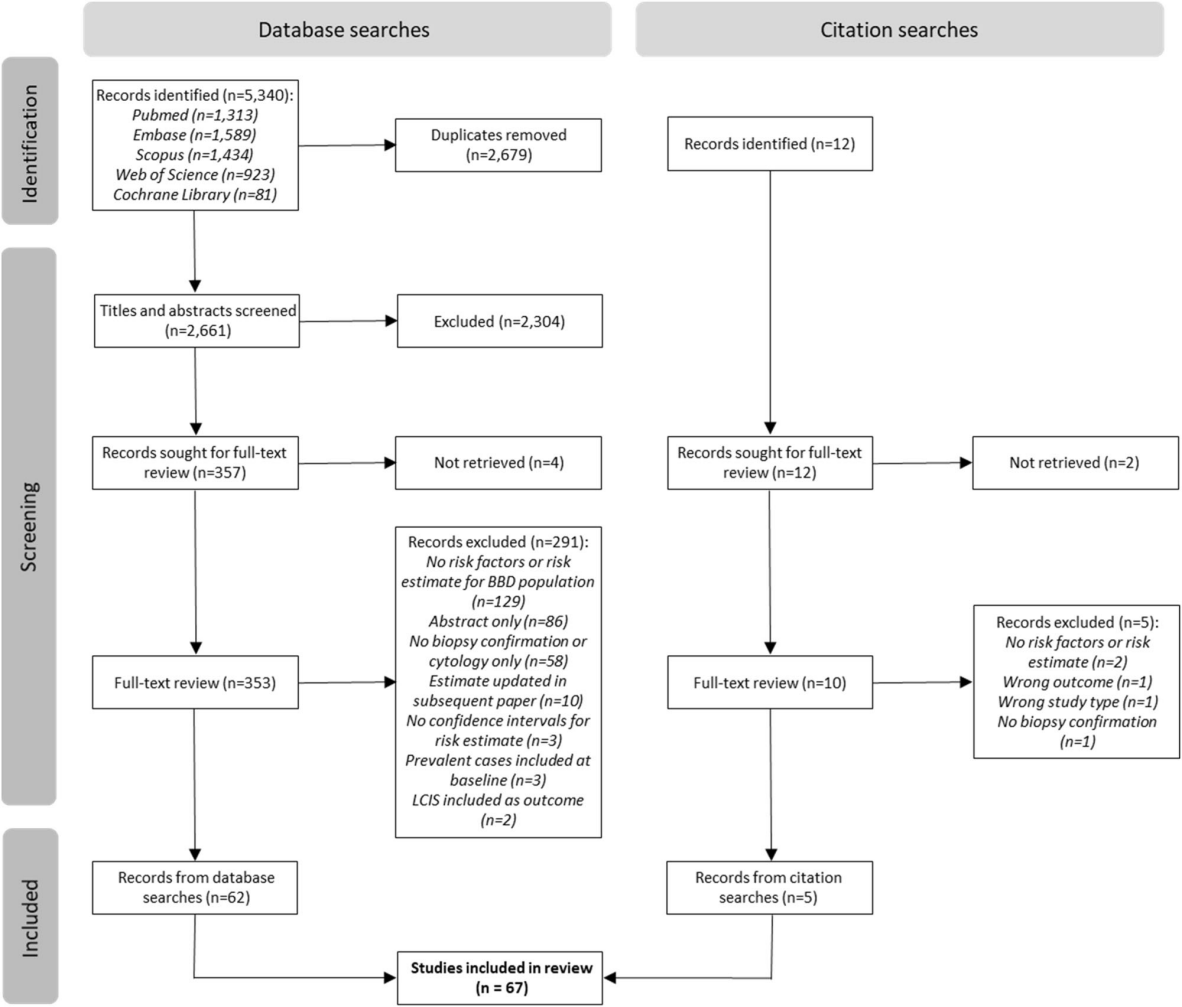
PRISMA flow diagram of study identification, screening and selection process.

The results of the database and reference searches are shown in the PRISMA flowchart in Fig. 1. A total of 5340 citations were retrieved from database searches and 12 additional citations from reference list searches. After removal of duplicates, non-English language studies, and ineligible publication and study types, the titles and abstracts of 2661 citations were screened, followed by full-text review of 363 studies, leading to 67 studies eligible for inclusion.

### Study characteristics

The characteristics of the included studies are presented in Supplementary Table 3. Of the 67 studies, 42 were cohort studies and 25 were case-control. Of the case-control studies, 17 were nested within larger cohorts. Just over half (*n* = 37) were population-based, with the remainder health-system or hospital-based (*n* = 30). Two studies used pooled data and were a combination of population-based and hospital-based. The majority of the studies were carried out in the United States (US; *n* = 56). Almost two-thirds (*n* = 44) were carried out in the mammographic era (post-1985 for US-based studies^35^) or as part of a screening study. The definition of breast cancer varied across studies: 23 included only diagnoses of invasive cancer, 30 included diagnoses of invasive or in situ carcinoma, and 14 did not specify. No minimum set of adjustment factors could be defined: the majority of studies adjusted for age (*n* = 56), with others adjusting for calendar period (*n* = 23), parity or number of pregnancies (*n* = 18), age at menarche (*n* = 18), family history of breast cancer (*n* = 19), or BMI (*n* = 12). One study presented unadjusted risk estimates^36^.

The risk factors evaluated in the included studies are presented in Supplementary Table 4. The most frequently evaluated were family history of breast cancer (*n* = 26) and age at biopsy (*n* = 24). Only risk factors identified in 4 studies or more are discussed in detail below. Risk factors assessed in 3 studies or fewer (Supplementary Table 4) included age at menopause (*n* = 3), bilateral oophorectomy (*n* = 3), previous benign biopsies (*n* = 3), use of NSAIDs (*n* = 2), duration of hormonal contraceptive use (*n* = 1), physical activity (*n* = 1), and use of chemoprevention (n = 1).

### Demographic factors and breast cancer risk among women with BBD

Among women with BBD, 26 studies found positive associations for age at biopsy and breast cancer risk (Tables 1 and 2). For any type of BBD, older age at biopsy was associated with up to 2.5-fold increased risk in two of three studies within BBD cohorts^33,37^ and in one that compared women with BBD to those without^38^ (Table 1). Three studies compared incidence of breast cancer among women with BBD to population-based incidence^18,39,40^, finding a similar magnitude of increased risk for all age groups (Table 1). Hartmann et al.^18^ reported decreasing strength of association with age compared to population-based incidence of breast cancer, in contrast to Said et al.^33^, from the same cohort, who found increased risk when comparing older to younger women with BBD.

**Table 1:** Associations of demographic factors and risk of breast cancer among women with BBD, for all BBD histologies combined




*BBD* benign breast disease, *BMI* body mass index, *HR* hazard ratio, *OR* odds ratio, *RR* relative risk, *SIR* standardised incidence ratio.

^a^Second-degree family history only.

^b^vs women without BBD and without family history.

^c^vs women without BBD within level of family history.

**Table 2:** Associations of demographic factors and risk of breast cancer among women with BBD, by BBD histological category





*AH* atypical hyperplasia, *ADH* atypical ductal hyperplasia, *ALH* atypical lobular hyperplasia, *BBD* benign breast disease, *BMI* body mass index, *HR* hazard ratio, *NPD* non-proliferative disease, *OR* odds ratio, *PDWA* proliferative disease without atypia, *RR* relative risk, *SIR* standardised incidence ratio.

^a^*vs*. BBD without radial scar.

^b^*vs*. women without BBD and without family history.

^c^*vs*. women without BBD within level of family history.

^d^*vs*. women without atypia and without family history.

Of the 17 studies that stratified by BBD histological category (Table 2), 13 found increasing age-associated risk, with the strongest associations among women with proliferative disease, with or without atypia^6,17,27,28,30–32,34,41–45^. Multiple risk estimates from the Mayo Clinic cohort are presented in Table 2. Pankratz et al.^9^ compared older to younger women with atypia, finding no association with age, while Hartmann et al. found 3.5–5.5-fold increased risk among women with atypia for all age groups in comparison to population-based rates^27^. Positive age-related risk associations were also reported from this cohort for fibroadenoma^30^ and sclerosing adenosis^34^, while mucocele-like lesions were associated with increased risk of breast cancer among women younger than 46 years at diagnosis^29^. We also identified multiple reports from the Nashville cohort. Compared to similarly-aged women with non-proliferative disease, increased risk was observed among women with proliferative disease without atypia aged 20–45 years at diagnosis^17^ and with lobular atypia for all age groups^32^. In comparison to population-based breast cancer incidence, age was positively associated with risk for proliferative diagnoses with or without atypia^17^ and ductal atypia^31^.

Seven studies evaluated the breast cancer risk associated with BMI among women with BBD, with no statistically significant associations reported (Tables 1 and 2). Only one study stratified by menopausal status, with findings suggestive of an inverse association for BMI among premenopausal women and a positive association among postmenopausal women^46^.

We identified 26 studies that examined the association of family history of breast cancer and BBD with breast cancer risk (Tables 1 and 2). In the majority of studies, family history was defined as first-degree (breast cancer in a mother, sister, or daughter). Dupont et al.^47^ additionally examined the association of second-degree family history (breast cancer in an aunt, grandmother, or half-sister). One study defined family history as breast cancer in a mother, sister, or grandmother^46^ and another did not include a definition^48^. For any type of BBD (Table 1), a 1.5- to 2-fold increase in risk was associated with the presence of family history^33,47,49^. Comparing women with BBD to non-BBD populations or to population-based incidence, similar or stronger associations for family history were reported^17,18,50–52^. In additional analyses within the Nashville cohort (Table 1), both first- and second-degree family history were associated with increased risk regardless of comparison group^17,47^.

Among the studies that reported risks by BBD histology (Table 2), family history was positively associated with risk regardless of comparison group. Additional analyses within the Nashville cohort showed that family history was associated with increased risk of breast cancer among women with sclerosing adenosis^51^ but not fibroadenoma^53^, and with proliferative findings with or without atypia, regardless of comparison group^17^ or whether the atypia was ductal or lobular^31^. Studies within the Mayo Clinic cohort (Table 2) reported a 4-fold increased risk associated with a strong family history, defined as breast cancer in more than one first-degree relative, among women with atypia^27^, fibroadenoma^30^, or sclerosing adenosis^34^ compared to population-based incidence of breast cancer; however, this association was not seen when comparing family history to no family history among women with atypia^9^.

Time since benign biopsy was positively associated with risk of breast cancer across the eight studies that evaluated it, regardless of comparison group (Tables 1 and 2). Increased risk was reported for at least 15 years post-biopsy for any type of BBD^38,40^ (Table 1), with the strongest associations among women with proliferative disease, with or without atypia^28,43,45,54,55^ (Table 2).

### Lifestyle factors and breast cancer risk among women with BBD

Six studies evaluated the association of alcohol consumption with risk of breast cancer among women with BBD (Table 3). Dupont et al.^47^ reported a stronger positive association among women with BBD who consumed alcohol (SIR 1.7, 95% CI 1.2–2.3) than those who did not (SIR 1.3, 95% CI 1.1–1.7) compared to population-based incidence of breast cancer, but not when comparing consumption to non-consumption within the same BBD cohort^47^. Among the remaining studies, no associations were reported, regardless of the comparison group, categorisation of alcohol consumption, or BBD histological category.

**Table 3:** Associations of lifestyle factors and risk of breast cancer among women with BBD, overall and by BBD histologic category

*AH* atypical hyperplasia, *BBD* benign breast disease, *HR* hazard ratio, *NPD* non-proliferative disease, *OR* odds ratio, *PDWA* proliferative disease without atypia, *RR* relative risk, *SIR* standardised incidence ratio.

^a^vs non-drinkers without atypia.

^b^vs never smokers without atypia.

Seven studies assessed the association between smoking and risk of breast cancer among women with BBD (Table 3). Dupont et al.^47^ found increased risk among never-smokers with BBD compared to population-based incidence of breast cancer, but not when comparing never-smokers with BBD to ever-smokers with BBD. No associations were reported in the remaining studies, regardless of comparison group, duration of smoking or of BBD histological category.

### Reproductive factors and breast cancer risk among women with BBD

The six studies that evaluated menarche-associated breast cancer risk were carried out within cohorts of women with BBD, with an additional comparison to population-based incidence of breast cancer in one study^47^. The direction of risk estimates indicated a possible inverse association for age at menarche for all BBD histologies combined (Table 4), with one study finding increased risk associated with menarche at age 12 or younger among women with BBD compared to population-based rates of breast cancer (SIR 1.5, 95% CI 1.10–2.00^47^). Of the two studies that presented associations by BBD histological category (Table 5), Tamimi et al.^56^ found a protective effect for later age at menarche among women with proliferative disease with (RR 0.83, 95% CI 0.73–0.93) or without atypia (RR 0.93, 95% CI 0.86–0.99), and an increased risk among women with non-proliferative lesions (RR 1.16, 95% CI 1.08–1.24).

**Table 4:** Associations of reproductive risk factors and risk of breast cancer among women with BBD, for all BBD histologies combined




*AFB* age at first birth, *BBD* benign breast disease, *EPT* oestrogen-progestin therapy, *HR* hazard ratio, *MHT* menopausal hormone therapy, *OR* odds ratio, *RR* relative risk, *SIR* standardised incidence ratio.

^a^among women with premenopausal BBD only.

**Table 5:** Associations of reproductive factors and risk of breast cancer among women with BBD, by BBD histologic category




*AFB* age at first birth, *AH* atypical hyperplasia, *ALH* atypical lobular hyperplasia, *BBD* benign breast disease, *EPT* oestrogen-progestin therapy, *HR* hazard ratio, *MHT* menopausal hormone therapy, *NPD* non-proliferative disease, *OR* odds ratio, *PDWA* proliferative disease without atypia, *RR* relative risk, *SIR* standardised incidence ratio.

^a^vs never users without BBD.

^b^vs no BBD and ≤55 years/premenopausal.

^c^among women with premenopausal BBD only.

^d^compared to never users within BBD histologic category.

We identified five studies that evaluated use of hormonal contraceptives on risk of breast cancer among women with BBD (Tables 4 and 5). In comparison to never users with or without BBD, a consistent inverse direction of risk estimates was reported for ever use, with one study^48^ finding a statistically significant association (OR 0.57, 95% CI 0.36–0.90; Table 4). In another^47^, never use, but not ever use, among women with BBD was associated with increased risk of breast cancer compared to population-based incidence rates (SIR 1.4, 95% CI 1.1–1.7); however, within the same cohort, never use compared to ever use among women with BBD was not associated with risk of breast cancer^47^ (Table 4).

Eight studies that evaluated parity-associated breast cancer risk among women with BBD were identified (Tables 4 and 5). Among women with any type of BBD (Table 4), Arthur et al. observed that parity was associated with reduced risk for up to two births, compared to those who were never pregnant^57^. Dupont et al.^58^ observed a 2-fold increased risk associated with nulliparity compared to women whose first birth was at or before age 20 (Table 4) and among nulliparous women with atypia compared to population-based incidence (Table 5). In the Mayo Clinic BBD cohort^59^, increased incidence of breast cancer was reported among nulliparous women with BBD compared to population-based rates (SIR 1.73, 95% CI 1.35–2.18). However, two additional studies from this cohort did not observe associations with breast cancer risk among parous compared to nulliparous women with any type of BBD (^60^, Table 4), or among nulliparous women with atypia compared to parous women aged 20-24 years at first birth^9^ (Table 5).

Eight studies presented associations for age at first birth with breast cancer risk among women with BBD (Tables 4 and 5), with inconsistent findings. For all BBD histologies (Table 4), two out of three studies found a protective effect for women who had younger ages at first birth compared to nulliparous women^61,62^. A further two studies compared older ages at first birth to younger ages among women with BBD, finding no association^58,63^, while Milanese et al.^59^ reported a positive risk association for women with BBD and first birth before age 30 years compared to population-based breast cancer incidence (SIR 1.27, 95% CI 1.13–1.41). Among the two studies that stratified by category of BBD histology (Table 5), no association with age at first birth among women with atypia was reported^9,64^, while in their additional comparison to population-based rates of breast cancer, Dupont et al.^58^, reported increased risk for first birth after age 20 among women with atypia (SIR 4.5, 95% CI 2.7–7.3).

For women with BBD of any type (Table 4), two of five studies showed an inverse association of menopausal status at biopsy with breast cancer risk for post- compared to premenopausal women^57,61^, while Worsham et al.^48^ found a positive association for the same comparison (OR 2.31, 95% CI 1.11–3.99). Hartmann et al.^18^ reported 1.5-fold increased risk among women with BBD, regardless of menopausal status, compared to population-based breast cancer incidence; however, this was not observed in a comparison of postmenopausal to premenopausal women within the same cohort^60^. Among the studies that examined risk by BBD histologic group (Table 5), premenopausal diagnoses of proliferative disease without atypia were associated with increased risk compared to premenopausal non-proliferative disease in two out of three studies^54,62^, while increased risk for women with atypia irrespective of menopausal status was reported in four out of five studies^32,54,62,65^, including the sub-cohort analysis of women with atypical lobular hyperplasia within the Nashville BBD cohort^32^. In comparison to premenopausal women without BBD, Ashbeck et al.^41^ observed increased breast cancer risk for both pre- and postmenopausal non-atypia diagnoses (Table 5).

Eleven studies assessed the association between MHT use and/or duration and risk of breast cancer among women with BBD (Tables 4 and 5). Two presented associations by type of MHT (combined oestrogen-progestin therapy or other^66,67^); the remainder did not differentiate by MHT formulation. Among women with BBD of any type (Table 4), six of eight studies reported no association for ever use or use of any duration compared to never use^48,60,61,67–69^ with risk of breast cancer. Compared to never users, Arthur et al. observed a 3.6-fold increased risk among ever users^57^, while Brinton et al.^70^ found increased risk associated with ten or more years of use following BBD diagnosis (RR 3.01, 95% CI 1.60–5.50), and a protective effect for ever use prior to BBD diagnosis (RR 0.60, 95% CI 0.40–0.90).

Three studies presented risk estimates by BBD histology (Table 5). Compared to never users with non-proliferative disease, Byrne et al. found that never and former MHT users with atypia were at 4-fold increased risk of breast cancer^71^; increased risk was also associated with fewer than five years’ use among women with proliferative diagnoses, with (RR 3.7, 95% CI 1.2–11.1) or without (RR 2.6, 95% CI 1.2–5.4) atypia. Lilleborge et al.^66^ reported increased risk for current combined therapy users with non-atypia diagnoses only (HR 1.96, 95% CI 1.27–3.02). Dupont et al.^68^ restricted their analysis to women who were diagnosed with BBD prior to menopause, observing increased risk among ever users with atypia compared to ever users with non-proliferative disease (RR 2.87, 95% CI 1.30–6.30). An earlier study in the same cohort^47^ found positive associations for women with atypia regardless of MHT use (never use: SIR 4.5, 95% CI 2.5–8.1; ever use: SIR 3.0, 95% CI 1.6–5.5), compared to population rates of breast cancer.

### Radiological factors and breast cancer risk among women with BBD

Seven studies examined the association of radiological or histological calcifications with risk of breast cancer among women with BBD (Tables 6 and 7). Among women with BBD of any type (Table 6), calcifications were associated with up to 3-fold increased risk regardless of comparison group across four studies^17,39,41,72^. Among the studies that presented risk estimates by BBD histology (Table 7), Hartmann et al.^27^ observed increased incidence of breast cancer among women with atypia irrespective of the presence of calcifications in comparison to population-based rates (no calcifications: SIR 4.63, 95% CI 3.31–6.31; calcifications: SIR 4.24, 95% CI 3.46–5.14). The two additional analyses from the Nashville cohort reported increased risk associated with calcifications among women with proliferative diagnoses, with or without atypia and regardless of comparison group^17^.

**Table 6:** Associations of radiological factors with risk of breast cancer among women with BBD, for all BBD histologies combined

*BBD* benign breast disease, *BI-RADS* Breast Imaging Reporting and Data System (I = almost entirely fat; II = scattered fibroglandular densities; III = heterogeneously dense; IV = extremely dense), *HR* hazard ratio, *MBD* mammographic breast density, *OR* odds ratio, *RR* relative risk, *SIR* standardised incidence ratio, *WPP* Wolfe’s parenchymal pattern (N1 = non-dense; P1 = ductal prominence occupying 25% of the breast; DY = extremely dense).

^a^vs no BBD and fatty/scattered densities.

**Table 7:** Associations of radiological factors with risk of breast cancer among women with BBD, by BBD histological category

*AH* atypical hyperplasia, *BBD* benign breast disease, *BI-RADS* Breast Imaging Reporting and Data System (I = almost entirely fat; II = scattered fibroglandular densities; III = heterogeneously dense; IV = extremely dense); *HR* hazard ratio, *MBD* mammographic breast density, *NPD* non-proliferative disease, *OR* odds ratio, *PDWA* proliferative disease without atypia, *RR* relative risk, *SIR* standardised incidence ratio.

^a^*vs*. Q1 within BBD histologic category.

^b^vs no BBD and fatty/scattered densities.

Among the 10 studies that evaluated MBD-associated breast cancer risk among women with BBD, consistent positive associations were reported (Tables 6 and 7). Across all comparison groups, higher MBD was associated with increased breast cancer risk, overall and by BBD category^15,19,21,22,36,41,60,73,74^.

### Quality appraisal

The classification of the included studies according to the relevant JBI quality appraisal and risk of bias tool^26^ is shown in Supplementary Table 7. Of 44 cohort studies, three failed to meet more than three criteria of the relevant checklist and were found to be at high risk of bias, 19 failed to meet two or three criteria and were deemed to be at moderate risk of bias. The remaining 22 cohort studies were found to be at low risk of bias. Of the 33 case-control studies, none were found to be at high risk of bias, 8 were deemed to be at moderate risk of bias, and the remaining 25 studies were deemed to be at low risk of bias.

## Discussion

We systematically reviewed the published literature for associations of demographic, lifestyle, reproductive, and radiological risk factors with risk of breast cancer among women with BBD. Findings from the included studies suggest positive risk factor associations for age, family history, nulliparity, calcifications and MBD among women with BBD, and no association for BMI, alcohol, smoking, and age at menarche with breast cancer risk in the manuscripts reviewed herein. Within the included literature, no clear patterns of association were observed for age at first birth, menopausal status at biopsy, and MHT use. We observed heterogeneity among the included studies in the areas of study time period, comparison groups, measurement of exposures, definition of outcomes, adjustment factors and measures of association.

Mammographic screening has increased the incidence of BBD^66,75^, especially small, non-palpable lesions^65^, and those containing atypia, diagnoses of which have approximately doubled since the introduction of mammography^18,57^. Furthermore, annual screening increases the likelihood of benign biopsy over a 10-year period compared to biennial or triennial screening intervals^76^. Estimates for the incidence of BBD vary by setting and purpose of mammogram: of 1.7 million breast biopsies performed per year in the United States for both screening and diagnostic purposes, approximately 70% are benign^77^. Data on the incidence of benign biopsies in European population-based screening settings is limited, but a study from the Norwegian breast screening programme suggests that approximately 50% of needle biopsies result in biopsy-confirmed benign diagnoses per year^66^. However the majority of women with a BBD diagnosis will not develop a future breast cancer^19,21^. An improved understanding of breast cancer risk among women biopsied for BBD may inform risk stratification approaches to prevention and early detection.

Of the demographic risk factors evaluated in this review, similar patterns of positive risk associations for family history and age were observed among women with BBD as among the general population^78^. However, age-related increases in breast cancer risk were strongest among women with proliferative disease, with or without atypia. Increased risk was sustained over a minimum of 10 years, highlighting the importance of monitoring risk over time and the continued surveillance of above-average-risk women^76^. Previous research among the general population has shown that elevated BMI increases risk of postmenopausal but not premenopausal breast cancer^78^, however, we identified only one study that presented risk associations for BMI by menopausal status^46^, limiting our ability to assess if a similar relationship with risk exists following a benign diagnosis.

Multiple studies^57–59,61,62^ suggested that among women with BBD, breast cancer risk associated with nulliparity reflects the positive association observed among the general population^79^. We did not identify clear patterns of risk associations for age at first birth, menopausal status, or MHT use among women with BBD, possibly reflecting the use of different comparison populations, referent groups and assessed confounders.

Among radiological risk factors, the identified literature consistently showed the independent association of MBD with increased breast cancer risk. Dense breasts in combination with atypia conferred the greatest risk in the included literature^6,19^. Calcifications were associated with increased breast cancer risk among women with BBD, irrespective of whether they were detected radiologically or histologically. However, the prognostic significance of calcifications associated with BBD remains unclear, as the studies presented here did not specify size or pattern of calcifications, features that may be indicative of higher-risk lesions^72^.

Many of the studies included in this review comprised white, US-based populations^17,18,62,80^, with a limited number evaluating BBD-associated risk among diverse populations^6,15,21,37,48,81,82^. As population-level distributions of breast cancer risk factors vary by region and race or ethnicity^78,83^, additional BBD research is needed that includes women from diverse populations to broaden understanding of aetiology and determinants of breast cancer risk and to better inform clinical management, prevention and early detection efforts across diverse populations.

The literature included in this review covers a broad time period that has seen widespread uptake of mammographic breast screening^35^ accompanied by advances in imaging technology and tissue sampling techniques^45^. While evidence suggests that estimates of breast cancer risk among women with BBD derived from pre-mammographic^35^ and surgical biopsy cohorts^3^ are comparable to those from contemporary cohorts, risk factor patterns have changed over this period. Compared to earlier cohorts, women diagnosed with BBD during the mammographic era are more likely to have higher BMI, older age at first birth and fewer total births^35,62,83^. In order to capture a comprehensive view of the literature, and as there are no clear temporal cut points for these changes in risk factor exposures, we chose not to restrict the time frame of the included studies. However, these secular changes may have contributed to variation in risk relationships in the included studies.

Breast cancer is a heterogeneous disease, and may be classified into subtypes according to expression of tumour markers such as the oestrogen receptor (ER^78^;). While BBD has been shown to be associated with increased risk of breast cancer irrespective of ER status^78^, prior studies suggest heterogeneity of risk factor associations, such as for parity or alcohol consumption, across tumour subtypes^78,79^. This may have contributed to differences in findings for some risk factors evaluated in this review. Only one of the studies identified as eligible for inclusion in this review evaluated risk of breast cancer among women with BBD by tumour subtype^62^. Future studies that account for tumour heterogeneity may help to clarify the aetiological factors that contribute to subtype-specific risk in this population.

As this review focussed on demographic, lifestyle, reproductive and radiological factors, we did not capture details on genetic determinants including polygenic risk scores within the identified literature. However, limited available evidence evaluating the role of polygenic risk scores among women with BBD suggests that polygenic risk scores and BBD are independent risk factors for breast cancer^84^. Improved knowledge of relevant risk factors for breast cancer among this population in combination with polygenic risk scores may improve efforts to stratify risk among women with BBD.

Strengths of this study include the systematic search process and the adherence to PRISMA guidelines for reporting^23^. This review was limited by heterogeneity of study time period, comparison groups, measurement of exposures, definition of outcomes, adjusting factors, measures of association, which precluded meta-analysis and presented challenges in narrative interpretation. We addressed this challenge by discussing the available data for each risk factor by BBD category (overall or by histology) and comparison group and highlighting sub-cohort analyses. The studies included were observational in design and may have been subject to systematic or random bias or low statistical power. We assessed the included studies for quality and risk of bias but did not exclude any studies on this basis in order to fully represent the existing literature. We acknowledge that the systematic review process was adapted in the areas of full-text review, data extraction and risk of bias assessment as they were carried out by a single reviewer; however, evidence suggests that simplification in these areas does not have a material impact on findings compared to a full systematic review^25^.

It must be acknowledged that many risk factors for BBD are also risk factors for breast cancer among the general population^4^. However, given the limited literature for many of the risk factors, we were unable to determine whether a risk factor may increase risk of breast cancer among all women (i.e. irrespective of a benign diagnosis), or by facilitating an environment for breast tissue changes, which may progress to breast cancer.

In summary, following a BBD diagnosis, risk of breast cancer appears to increase with age at diagnosis, nulliparity, dense breasts, and family history of breast cancer. Characterising determinants of risk in this population is challenging as the studies to date have been carried out over a period that has seen the introduction of mammographic screening and secular changes in reproductive and demographic risk factor patterns. Improved awareness of factors that increase breast cancer risk among this population may inform clinical management, prevention or early detection strategies. Future studies and consortial efforts accounting for racial and ethnic diversity and heterogeneity in BBD and breast cancer diagnoses are needed to further understand determinants of risk in this population.

## Supplementary information

Supplementary data

## Data Availability

The data included in this systematic review were collated from published studies identified through searches of electronic literature databases PubMed, Scopus, Embase, Web of Science and the Cochrane Library using the search strategies in Supplementary Table 2.
