# Supplementary material for: A systematic review of determinants of breast cancer risk among women with benign breast disease
Source: NPJ Breast Cancer. 2025 Feb 15;11:16. doi: 10.1038/s41523-024-00703-w (PMC11829998; doi:10.1038/s41523-024-00703-w)
Supplement: Supplementary file 1 — Supplementary data [file 41523_2024_703_MOESM1_ESM.pdf]

Determinants of breast cancer risk among women with benign breast disease: a systematic review

Supplementary Data

1. Supplementary Table 1: PRISMA checklist
2. Supplementary Table 2: Search strategy
3. Supplementary Table 3: Characteristics of studies identified as eligible for inclusion
4. Supplementary Table 4: Risk factors evaluated in the included studies
5. Supplementary Table 5: Joanna Briggs Institute Critical Appraisal Checklist for Cohort Studies
6. Supplementary Table 6: Joanna Briggs Institute Critical Appraisal Checklist for Case-Control Studies
7. Supplementary Table 7: Quality appraisal and risk of bias assessment

Supplementary Table 1: PRISMA checklist

Section and Topic	Item #	Checklist item	Location where item is reported
**TITLE**			
Title	1	Identify the report as a systematic review.	p. 1
**ABSTRACT**			
Abstract	2	See the PRISMA 2020 for Abstracts checklist.	p. 2
**INTRODUCTION**			
Rationale	3	Describe the rationale for the review in the context of existing knowledge.	p. 3
Objectives	4	Provide an explicit statement of the objective(s) or question(s) the review addresses.	p. 3
**METHODS**			
Eligibility criteria	5	Specify the inclusion and exclusion criteria for the review and how studies were grouped for the syntheses.	p. 4
Information sources	6	Specify all databases, registers, websites, organisations, reference lists and other sources searched or consulted to identify studies. Specify the date when each source was last searched or consulted.	p. 4
Search strategy	7	Present the full search strategies for all databases, registers and websites, including any filters and limits used.	Supplementary Table 2
Selection process	8	Specify the methods used to decide whether a study met the inclusion criteria of the review, including how many reviewers screened each record and each report retrieved, whether they worked independently, and if applicable, details of automation tools used in the process.	p. 4
Data collection process	9	Specify the methods used to collect data from reports, including how many reviewers collected data from each report, whether they worked independently, any processes for obtaining or confirming data from study investigators, and if applicable, details of automation tools used in the process.	p. 5
Data items	10a	List and define all outcomes for which data were sought. Specify whether all results that were compatible with each outcome domain in each study were sought (e.g. for all measures, time points, analyses), and if not, the methods used to decide which results to collect.	p. 5
	10b	List and define all other variables for which data were sought (e.g. participant and intervention characteristics, funding sources). Describe any assumptions made about any missing or unclear information.	p. 5
Study risk of bias assessment	11	Specify the methods used to assess risk of bias in the included studies, including details of the tool(s) used, how many reviewers assessed each study and whether they worked independently, and if applicable, details of automation tools used in the process.	p. 5
Effect measures	12	Specify for each outcome the effect measure(s) (e.g. risk ratio, mean difference) used in the synthesis or presentation of results.	p. 5
Synthesis methods	13a	Describe the processes used to decide which studies were eligible for each synthesis (e.g. tabulating the study intervention characteristics and comparing against the planned groups for each synthesis (item #5)).	p. 5
	13b	Describe any methods required to prepare the data for presentation or synthesis, such as handling of missing summary statistics, or data conversions.	p. 5
	13c	Describe any methods used to tabulate or visually display results of individual studies and syntheses.	p. 5
	13d	Describe any methods used to synthesize results and provide a rationale for the choice(s). If meta-analysis was performed, describe the model(s), method(s) to identify the presence and extent of statistical heterogeneity, and software package(s) used.	p. 5
	13e	Describe any methods used to explore possible causes of heterogeneity among study results (e.g. subgroup analysis, meta-regression).	N/A
	13f	Describe any sensitivity analyses conducted to assess robustness of the synthesized results.	N/A
Reporting bias assessment	14	Describe any methods used to assess risk of bias due to missing results in a synthesis (arising from reporting biases).	p. 5

Section and Topic	Item #	Checklist item	Location where item is reported
Certainty assessment	15	Describe any methods used to assess certainty (or confidence) in the body of evidence for an outcome.	N/A
**RESULTS**			
Study selection	16a	Describe the results of the search and selection process, from the number of records identified in the search to the number of studies included in the review, ideally using a flow diagram.	Figure 1
	16b	Cite studies that might appear to meet the inclusion criteria, but which were excluded, and explain why they were excluded.	p. 7
Study characteristics	17	Cite each included study and present its characteristics.	Supplementary Table 3
Risk of bias in studies	18	Present assessments of risk of bias for each included study.	Supplementary Table 7
Results of individual studies	19	For all outcomes, present, for each study: (a) summary statistics for each group (where appropriate) and (b) an effect estimate and its precision (e.g. confidence/credible interval), ideally using structured tables or plots.	Tables 1-7
Results of syntheses	20a	For each synthesis, briefly summarise the characteristics and risk of bias among contributing studies.	Supplementary Table 7
	20b	Present results of all statistical syntheses conducted. If meta-analysis was done, present for each the summary estimate and its precision (e.g. confidence/credible interval) and measures of statistical heterogeneity. If comparing groups, describe the direction of the effect.	N/A
	20c	Present results of all investigations of possible causes of heterogeneity among study results.	p. 7
	20d	Present results of all sensitivity analyses conducted to assess the robustness of the synthesized results.	N/A
Reporting biases	21	Present assessments of risk of bias due to missing results (arising from reporting biases) for each synthesis assessed.	N/A
Certainty of evidence	22	Present assessments of certainty (or confidence) in the body of evidence for each outcome assessed.	N/A
**DISCUSSION**			
Discussion	23a	Provide a general interpretation of the results in the context of other evidence.	p. 14-17
	23b	Discuss any limitations of the evidence included in the review.	p. 16
	23c	Discuss any limitations of the review processes used.	p. 16
	23d	Discuss implications of the results for practice, policy, and future research.	p. 17
**OTHER INFORMATION**			
Registration and protocol	24a	Provide registration information for the review, including register name and registration number, or state that the review was not registered.	p. 4
	24b	Indicate where the review protocol can be accessed, or state that a protocol was not prepared.	p. 4
	24c	Describe and explain any amendments to information provided at registration or in the protocol.	p. 4
Support	25	Describe sources of financial or non-financial support for the review, and the role of the funders or sponsors in the review.	p. 17
Competing interests	26	Declare any competing interests of review authors.	p. 1
Availability of data, code and other materials	27	Report which of the following are publicly available and where they can be found: template data collection forms; data extracted from included studies; data used for all analyses; analytic code; any other materials used in the review.	p.17; Supplementary data

From: Page MJ, McKenzie JE, Bossuyt PM, Boutron I, Hoffmann TC, Mulrow CD, et al. The PRISMA 2020 statement: an updated guideline for reporting systematic reviews. BMJ 2021;372:n71. doi: 10.1136/bmj.n71

Supplementary Table 2: Search strategy

Database	Search string
PubMed	("Breast Neoplasms"[Mesh] AND "Risk"[Mesh] AND ("Breast Diseases"[Mesh] OR "Fibrocystic Breast Disease"[Mesh] OR "Hyperplasia"[MeSH]) AND ("benign breast disease" OR proliferative OR atypi* OR "benign lesion" OR "benign biopsy")) OR ((("benign breast disease*" OR "benign lesion" OR "premalignant lesion" OR "benign biopsy") AND ("risk factor*"[Title/Abstract] OR "risk association*"[Title/Abstract] OR "risk profile*"[title/abstract] OR "risk of breast cancer"[title/abstract])) NOT medline[sb])
Embase	'breast tumor'/exp AND ('breast disease'/exp OR 'fibrocystic breast disease'/exp OR 'hyperplasia'/exp) AND ('benign breast disease':ab,ti OR proliferative:ab,ti OR atypi*:ab,ti OR 'benign lesion':ab,ti OR 'benign biopsy':ab,ti OR 'premalignant lesion':ab,ti) AND ('risk factor*':ab,ti OR 'risk association*':ab,ti OR 'risk profile*':ab,ti OR 'risk of breast cancer':ab,ti)
Web of Science	(((TS=(Breast cancer))) AND AB=(("benign breast disease" OR proliferative OR atypi* OR "benign lesion" OR "benign biopsy" OR "premalignant lesion"))) AND AB=(("risk factor*" OR "risk association*" OR "risk profile*" OR "risk of breast cancer"))
Scopus	(TITLE-ABS-KEY ({breast cancer}) AND TITLE-ABS-KEY ({benign breast disease} OR proliferative OR atypi* OR {benign lesion} OR {benign biopsy} OR {premalignant lesion}) AND TITLE-ABS-KEY ({risk factor} OR {risk association} OR {risk profile} OR {risk of breast cancer}))
Cochrane Library	Breast cancer AND ("benign breast disease" OR proliferative OR atypi* OR "benign lesion" OR "benign biopsy" OR "premalignant lesion") AND ("risk factor*" OR "risk association*" OR "risk profile*" OR "risk of breast cancer") AND ("benign breast disease" OR proliferative OR atypi* OR "benign lesion" OR "benign biopsy" OR "premalignant lesion") AND ("risk factor*" OR "risk association*" OR "risk profile*" OR "risk of breast cancer")

Supplementary Table 3: Characteristics of studies identified as eligible for inclusion (n=67).

First author, year	Study, location	Duration of study	Study setting and design	Sample size	Age at BBD diagnosis	Outcome	Risk factors examined	Covariates included in final model
Abubakar *et al,* 2021 (73)	Kaiser Permanente Northwest (KPNW), USA	1970-2012	Hospital-based, nested case-control	1,028	Median age, years = 51.5	Invasive carcinoma	MBD	Age at menarche, parity/age at FFTB, BMI, menopausal status/MHT use, bilateral oophorectomy, first-degree FH, BBD histology, lobular involution, year of BBD diagnosis, MBD, age at BBD diagnosis, follow-up duration, epithelium-to-stroma proportion
Aroner *et al,* 2013 (28)	Nurses' Health Study (NHS) & Nurses' Health Study II (NHS-II), USA	1976-1998 (NHS) 1989-1995 (NHS II)	Population-based, nested case-control	2,252	Mean age, years = 44.6	Invasive or in situ carcinoma	Age at BBD diagnosis, time since BBD diagnosis	Age, year of BBD diagnosis, time since BBD biopsy, BMI at age 18, weight change since age 18, FH, age at menarche, parity/age at FFTB, BBD histology
Arthur *et al,* 2017 (57)	KPNW, USA	1971-2006	Hospital-based, nested case-control	1,052	NR	Invasive carcinoma	Smoking, BMI, FH, age at menarche, age at FFTB, number of pregnancies, menopausal status at BBD diagnosis, MHT use, duration of MHT use	Cigarette smoking status, pack-years of cigarette smoking, BMI, first-degree FH, age at menarche, age at FFTB, number of pregnancies, oophorectomy, MHT use, duration of MHT use, BBD histology, lobular involution
Ashbeck *et al,* 2007 (41)	New Mexico Mammography Project, USA	1992-2000	Population-based, retrospective cohort	229,885	Aged <50 years: no BBD = 47.4% BBD = 53.7%	Invasive or in situ carcinoma	Menopausal status at BBD diagnosis, MBD, FH	Age
Bodian *et al,* 1993 (49)	USA	1930-1982	Hospital-based, retrospective cohort	1,799	Mean age, years = 42.6	Invasive or in situ carcinoma	FH	Age, year of entry biopsy, length of follow-up
Brinton *et al,* 1986(70)	BCDDP, USA	1973-1980	Population-based, nested case-control	4,218	NR	Invasive or in situ carcinoma	Duration of MHT use	Age, type of menopause, interval since oophorectomy
Buckley *et al,* 2015 (42)	BreastScreen South Australia, Australia	1989-2010	Population-based, retrospective cohort	272,047	Median age, years: ADH = 54 ALH = 55	Invasive or in situ carcinoma	Age at BBD diagnosis, time since BBD diagnosis	SES, remoteness of residence, baseline age*time, year of first screen
Byrne *et al,* 1991(50)	BCDDP, USA	1973-1980	Population-based, nested case-control	6,088	NR	Invasive carcinoma; NR if carcinoma in situ included	FH	Age at diagnosis
Byrne *et al,* 2000 (71)	NHS, USA	1976-1992	Population-based, nested case-control	743	Mean age, years: NPD = 46.4 PDWA = 46.9 AH = 50.9	Postmenopausal invasive or in situ carcinoma	Menopausal status at BBD diagnosis, MHT use, duration of MHT use	Year of birth, year of BBD diagnosis, age at menarche, parity, age at FFTB, FH, menopause type, age at menopause, BMI
Byrne *et al,* 2001 (15)	BCDDP, USA	1973-1980	Population-based, nested case-control	757	Mean age, years: cases = 59.5 controls = 59.5	Invasive carcinoma; NR if carcinoma in situ included	MBD	Age, race, FH, alcohol, nulliparity, age at FFTB, years of education, weight at study entry, menopausal status, age at menopause, use of MHT
Castells *et al,* 2015 (75)	Benign Lesion (BELE) Study, Spain	1994-2011	Population-based, retrospective cohort	413,873	Aged 50-54 years: no BBD = 55.3% NPD = 47.9% PDWA = 46.6% AH = 49.7%	Invasive or in situ carcinoma	FH	Age at screen

Collins *et al*, 2006 (16)	NHS & NHS-II, USA	1976-1996 (NHS) 1989-1995 (NHS II)	Population-based, nested case-control	2,005	mean age, years cases = 43.5, controls = 43.8	Invasive or in situ carcinoma	FH	Age at BC diagnosis or index date, year of BBD diagnosis, length of follow-up, age at menarche, parity/age at FFTB, BMI, menopausal status/type of menopause, status/duration of MHT use
Collins *et al,* 2007 (54)	NHS & NHS-II, USA	1976-1996 (NHS) 1989-1995 (NHS II)	Population-based, nested case-control	2,005	Mean age, years cases = 43.5, controls = 43.8	Invasive or in situ carcinoma	Menopausal status at BBD diagnosis, time since BBD diagnosis	Age at BC diagnosis or index date, year of BBD diagnosis, length of follow-up, FH, age at menarche, parity/age at FFTB, BMI, menopausal status/type of menopause, status/duration of MHT use
Cote *et al,* 2012 (86)	USA	1997-2000	Population-based, retrospective cohort	1,406	Mean age, years = 48.6	Invasive or in situ carcinoma	Age at BBD diagnosis	Age, year of BBD diagnosis
Dupont *et al,* 1985 (17)	Nashville BBD cohort, USA	1950-1968	Hospital-based, retrospective cohort	3,303	Mean age, years = 42	Invasive carcinoma	Age at BBD diagnosis, FH	Age at entry
Dupont *et al,* 1987 (58)	Nashville BBD cohort, USA	1950-1968	Hospital-based, retrospective cohort	3,303	Mean age, years = 42	Invasive carcinoma	Age at FFTB	Age at BBD diagnosis, year of BBD diagnosis, length of follow-up
Dupont *et al,* 1989(47)	Nashville BBD cohort, USA	1950-1968	Hospital-based, retrospective cohort	3,303	Mean age, years = 42	Invasive carcinoma	Alcohol, age at menarche, age at menopause, FH, HC use, MHT use, smoking	Age at BBD diagnosis, year of BBD diagnosis, length of follow-up
Dupont *et al,* 1993 (65)	BCDDP, USA	1973-1978	Population-based, nested case-control	322	NR	Invasive carcinoma	FH	Study centre, age at BBD diagnosis, year of BBD diagnosis, parity
Dupont *et al,* 1994 (53)	Nashville BBD cohort, USA	1950-1968	Hospital-based, retrospective cohort	1,835	Mean age, years: BBD = 31.8, no BBD = 31.2	Invasive carcinoma	FH	Age at BBD diagnosis, length of follow-up, parity, age at FFTB, age at menarche
Dupont *et al,* 1999 (68)	Nashville BBD cohort, USA	1952-1978	Hospital-based, retrospective cohort	5,813	NR	Invasive carcinoma	MHT use	Age, year of BBD diagnosis, length of follow-up, parity
Figueroa *et al,* 2021 (62)	KPNW, USA	1971-2006	Hospital-based, nested case-control	1,028	Median age, years = 51.5	Invasive carcinoma	FH	BBD diagnosis year, age at BBD diagnosis, duration of membership of health plan, length of follow-up, first-degree FH, history of bilateral oophorectomy, parity
Gail *et al,* 2007 (90)	CARE Study, USA	1994-1998	Population-based, case-control	3,254	NR	Invasive carcinoma	Number of benign biopsies by age	Age at menarche, number of benign biopsies, FH
Gallicchio *et al,* 2006 (46)	CLUE-II, USA	1989-1996	Population-based, retrospective cohort	1,467	37.2% of cohort aged <50 years	Invasive or in situ carcinoma	BMI, FH, age at FFTB, parity, age at menarche, smoking, alcohol consumption, NSAID use	Age
Ghosh *et al,* 2010 (60)	Mayo Clinic BBD Cohort, USA	1967-1981	Hospital-based, retrospective cohort	2,666	Mean age, years = 54.7	Invasive carcinoma; NR if carcinoma in situ included	BMI, parity, MHT use, menopausal status at BBD diagnosis, MBD	Age. Parenchymal pattern models also adjusted for BBD histology, BMI, parity, menopausal status, lobular involution.
Hartmann *et al,* 2005 (18)	Mayo Clinic BBD Cohort, USA	1967-1981	Hospital-based, retrospective cohort	9,087	Mean age, years = 51.4	Invasive carcinoma; NR if carcinoma in situ included	Age at BBD diagnosis, menopausal status at BBD diagnosis, FH	Age, calendar period

Hartmann *et al,* 2014 (27)	Mayo Clinic BBD Cohort, USA	1967-2001	Hospital-based, retrospective cohort	698	47.7% of cohort aged <55 years	Invasive or in situ carcinoma	Age at BBD diagnosis, FH	Age, calendar period
Horn-Ross *et al,* 2004 (81)	California Teachers Study, USA	1995-1996	Population-based, prospective cohort	103,460	NR	Invasive carcinoma	Alcohol consumption	Age, race/ethnicity, caloric intake, age at menarche, FH, BMI, nulliparity/age at FFTP, physical activity, duration of MHT use
Hutchinson *et al,* 1980 (39)	USA	1940-1975	Population-based, prospective cohort	1,441	Mean age, years = 41	Invasive carcinoma	Age at BBD diagnosis	Age, calendar year
Jensen *et al,* 1989 (51)	Nashville BBD cohort, USA	1950-1968	Hospital-based, retrospective cohort	313	NR	Invasive carcinoma	FH	Age
Kabat *et al,* 2010a (43)	Multi-centre (Canada, USA, UK)	Canada: 1980-1988; UK: 1946-1984; USA: 1970-1994	Population-based (Canada), hospital-based (UK, USA), nested case-control	1,239	Mean age, years = 48.7	Invasive or in situ carcinoma	Age at BBD diagnosis, time since BBD diagnosis	Age at menarche, age at FFTB, number of pregnancies, menopausal status, first-degree FH
Kabat *et al,* 2010b (61)	Multi-centre (Canada, USA, UK)	Canada: 1980-1988; UK: 1946-1984; USA: 1970-1994	Population-based (Canada), hospital-based (UK, USA), nested case-control	1,357	NR	Invasive or in situ carcinoma	Age at FFTB/number of pregnancies, FH, menopausal status at BBD diagnosis, BMI, HC use, HC duration, MHT use, smoking	Age at FFTB/number of pregnancies, FH, menopausal status, BBD histology
Kilgore *et al,* 2021 (82)	USA	2004-2018	Population-based, retrospective cohort	387	NR	Invasive or in situ carcinoma	Age at BBD diagnosis, chemoprevention	Age, race, chemoprevention, surgical excision of ADH, imaging modality used to guide biopsy
Krieger *et al,* 1992 (40)	Kaiser Permanente Oakland BBD cohort, USA	1948-1973	Hospital-based, retrospective cohort	2,731	Mean age, years = 38.3	Invasive carcinoma; NR if carcinoma in situ included	Age at BBD diagnosis, time since BBD diagnosis	Age
Lilleborge *et al,* 2021 (66)	BreastScreen Norway, Norway	2006-2016	Population-based, retrospective cohort	14,621	Median age, years: benign lesion = 54 AH = 57	Invasive carcinoma	Physical activity, BMI, alcohol, smoking, MHT use	Age, physical activity, BMI, acohol consumption, smoking, MHT use
Lubin *et al,* 1983 (38)	Canada	1976-1977	Population-based, case-control	1,403	NR	Invasive carcinoma; NR if carcinoma in situ included	Age at BBD diagnosis, time since BBD diagnosis	Age
Magnusson *et al,* 1998 (85)	Sweden	1993-1995	Population-based, case-control	5,990	Mean age, years: cases = 62.0, controls = 62.9	Invasive carcinoma	FH	Age, age at menarche, parity/age at FFTB, age at menopause, menopausal status, use of MHT or HC >1 year, height
Marshall *et al,* 1997 (55)	NHS, USA	1976-1988	Population-based, nested case-control	856	Median age, years = 43	Invasive or in situ carcinoma	Time since BBD diagnosis	Year of benign biopsy, age at menarche, parity, age at FFTB, FH, menopausal status
McCredie *et al,* 1997 (52)	New Zealand	1983-1987	Population-based, case-control	2,755	Aged <50 years: cases = 74.5% controls = 84.2%	Invasive carcinoma	FH	Age, ethnicity, parity, age at menarche
McDivitt *et al,* 1992 (87)	CDC CHCS, USA	1980-1982	Population-based, case-control	8,044	NR	Invasive carcinoma; NR if carcinoma in situ included	FH	Age, age at menarche, age at menopause, age at FFTB
Meares *et al,* 2016 (29)	Mayo Clinic BBD Cohort, USA	1967-2001	Hospital-based, retrospective cohort	13412	33% of the cohort aged <45 years	Invasive carcinoma; NR	Age at BBD diagnosis	Age, calendar period

						if carcinoma in situ included		
Milanese *et al*, 2006 (59)	Mayo Clinic BBD Cohort, USA	1967-1991	Hospital-based, retrospective cohort	8736	46% of the cohort aged <50 years	Invasive carcinoma; NR if carcinoma in situ included	Age at FFTB	Age, calendar period
Moon *et al*, 2014 (44)	Korea	2000-2010	Hospital-based, retrospective cohort	12,302	Mean age, years = 45.3	Invasive carcinoma; NR if carcinoma in situ included	Age at BBD diagnosis	Age, FH, length of follow-up, MBD
Nassar *et al*, 2015 (30)	Mayo Clinic BBD Cohort, USA	1967-1991	Hospital-based, retrospective cohort	9,076	Mean age, years: no FA = 52.2 simple FA = 45.8 complex FA = 50.2	Invasive carcinoma; NR if carcinoma in situ included	Age at BBD diagnosis, FH	Age, calendar period of diagnosis
Newcomb *et al*, 1995 (69)	USA	1989-1991	Population-based, case-control	6828	NR	Invasive carcinoma	MHT use	Type of menopause, time since menopause, age at menarche, age at FFTB, BMI, FH, alcohol consumption, education
Page *et al*, 1985 (31)	Nashville BBD cohort, USA	1950-1968	Hospital-based, retrospective cohort	268	Mean age, years = 46	Invasive carcinoma	Age at BBD diagnosis, FH	Age, number of years at risk
Page *et al*, 2003 (32)	Nashville BBD cohort, USA	1950-1985	Hospital-based, retrospective cohort	252	36% of cohort aged ≤45 years	Invasive carcinoma	Age at BBD diagnosis, approximate menopausal status at BBD diagnosis	Age at BBD diagnosis
Pankratz *et al*, 2008 (9)	Mayo Clinic BBD Cohort, USA	1967-1991	Hospital-based, retrospective cohort	331	Mean age, years = 58	Invasive carcinoma	Age at BBD diagnosis, age at menarche, age at FFTB, FH, number of biopsies	Age at BBD diagnosis, age at menarche, age at FFTB, first-degree FH, number of benign biopsies
Posso *et al*, 2022 (72)	BELE Study Group, Spain	1995-2015	Population-based, retrospective cohort	10,650	Mean age, years = 56	Invasive or in situ carcinoma	Age	Age, calendar year
Román *et al*, 2021 (19)	BELE Study Group, Spain	1995-2015	Population-based, retrospective cohort	294,943	64% of cohort aged <55 years	Invasive or in situ carcinoma	MBD	Age at screen, year of screen
Román *et al*, 2022 (45)	BELE Study Group, Spain	1995-2015	Population-based, retrospective cohort	778,306	50.7% of the cohort aged <55 years	Invasive or in situ carcinoma	Age at index mammogram, time since index mammogram	Year, age, time since mammogram
Said *et al*, 2015(33)	Mayo Clinic BBD Cohort, USA	1967-2001	Hospital-based, retrospective cohort	11,591	33% of cohort aged <45 years	Invasive or in situ carcinoma	Age at BBD diagnosis, FH	Flat epithelial atypia, BBD histology, age at BBD diagnosis, year of BBD diagnosis, lobular involution, FH
Schonberg *et al*, 2016 (91)	NHS, Women's Health Initiative (WHI), USA	2004-2006 (NHS) 2005-2010 (WHI)	Population-based, retrospective cohort	170,153	Mean age, years: NHS = 70 WHI = 71	Invasive carcinoma	Number of benign biopsies	Age at menarche, age at FFTB*first-degree FH
Sherman *et al*, 2020 (92)	Mayo Clinic BBD Cohort, USA	1992-2001	Hospital-based, retrospective cohort	3,080	Mean age, years = 50.3	Invasive or in situ carcinoma	NSAID use	Age at BBD diagnosis, BBD histology, lobular involution, BMI, years from BBD diagnosis to questionnaire completion
Stanford *et al*, 1995 (67)	USA	1988-1990	Population-based, case-control	1029	Aged <55 years: cases = 28.3% controls = 30.9%	Invasive or in situ carcinoma	MHT use	Age, age at FFTB, FH
Tamimi *et al*, 2005 (88)	NHS & NHS-II, USA	1976-1998 (NHS) 1989-1999 (NHS II)	Population-based, nested case-control	1505	Mean age among controls, years: NPD = 42.5 PDWA = 43.7 AH = 50.1	Invasive or in situ carcinoma	Alcohol consumption	Age at BC diagnosis or index date, year of benign BBD diagnosis, length of follow-up, age at menarche, parity/age at FFTB, BMI, FH, menopausal status/type

								of menopause, status/duration of MHT use
Tamimi *et al*, 2010 (56)	NHS & NHS-II, USA	1976-2000	Population-based, prospective cohort	75,022	NR	Invasive or in situ carcinoma	Age at menarche, age at menopause	NR
Thomas *et al*, 1982 (63)	USA	1942-1975	Hospital-based, retrospective cohort	1,353	NR	Invasive carcinoma	Age at menarche, age at FFTB, menopausal status at BBD diagnosis	Age at BBD diagnosis, age at follow-up
Tice *et al*, 2013 (21)	Breast Cancer Surveillance Consortium (BCSC), USA	1994-2009	Population-based, prospective cohort	42,818	Median age, years = 52.2	Invasive or in situ carcinoma	MBD	Age, race/ethnicity, study site
Tice *et al*, 2015(6)	BCSC, USA	1994-2010	Population-based, prospective cohort	1,135,977	49% of cohort aged <50 years	Invasive carcinoma	Age at BBD diagnosis	Age, race/ethnicity
Vierkant *et al*, 2017 (22)	Mayo Clinic BBD Cohort, USA	1985-2001	Hospital-based, retrospective cohort	6,271	NR	Invasive carcinoma; NR if carcinoma in situ included	MBD	Age, calendar period
Visscher *et al*, 2014 (34)	Mayo Clinic BBD Cohort, USA	1967-2001	Hospital-based, retrospective cohort	13,434	32.6% of cohort aged <45 years	Invasive or in situ carcinoma	Age at BBD diagnosis, FH	Age, calendar period
Whiffen *et al*, 2011 (64)	USA	1991-?	Hospital-based, retrospective cohort	1,598	Median age, years: AH = 60, without AH = 61	Invasive carcinoma; NR if carcinoma in situ included	FH, use of HC, BMI, alcohol consumption, smoking, age at FFTB	Atypia
Worsham *et al*, 2007 (37)	USA	1981-1994	Hospital-based, retrospective cohort	4,465	63% of cohort aged <50 years	Invasive or in situ carcinoma	Age at BBD diagnosis	Age, fibrosis, multiplicity of benign lesions
Worsham *et al*, 2009 (48)	USA	1981-1994	Hospital-based, nested case-control	872	Aged <50 years: cases = 46% controls = 63%	Invasive or in situ carcinoma	MHT use, HC use, menopausal status at BBD diagnosis	Date of BBD diagnosis, BBD histological type, FA, race*age
Yaghjyan *et al*, 2015 (74)	NHS, USA	1989-2004	Population-based, nested case-control	2,838	Mean age at mammogram, years: cases = 60.2 controls = 60.7	Invasive or in situ carcinoma	MBD	Age at initial blood draw, menopausal status, MHT use at blood draw, day/time of blood draw
Yoon *et al*, 2020 (36)	Korea	2003-2014	Hospital-based, retrospective cohort	205	Mean age, years = 47.1	Invasive or in situ carcinoma	Age at BBD diagnosis, MBD	Unadjusted
CDC CSHS, 1983 (89)	USA	1980-1981	Population-based, case-control	1,766	Aged <50 years: cases = 68.7% controls = 67.5%	Invasive carcinoma; NR if carcinoma in situ included	HC use	Age

AH = atypical hyperplasia; BBD = benign breast disease; BC = breast cancer; BMI = body mass index; FA = fibroadenoma; FFTB = first full-term birth; FH = family history of breast cancer; HC = hormonal contraceptive; MBD = mammographic breast density; MHT = menopausal hormone therapy; NPD = non-proliferative disease; NR = not reported; PDWA = proliferative disease without atypia; SES = socioeconomic status

Supplementary Table 4: Risk factors evaluated in the included studies (n=67).

	Demographic risk factors					*Lifestyle risk factors*		*Reproductive risk factors*						*Radiological risk factors*		*Not assessed further*						
First author, year	Age at BBD diagnosis	BMI	Family history	Race/ethnicity	Time since biopsy	Alcohol	Smoking	Age at menarche	Hormonal contraceptive use	Parity	Age at first birth	Menopausal status at biopsy	MHT use and duration	Calcifications	MBD	Age at menopause	Bilateral oophorectomy	Previous benign biopsies	NSAID use	Chemoprevention	Hormonal contraceptive duration	Physical activity
Abubakar et al, 2021 (73)															•							
Aroner et al, 2013 (28)	•				•																	
Arthur et al, 2017 (57)		•					•	•		•		•	•									
Ashbeck et al, 2007 (41)	•		•									•		•	•							
Bodian et al, 1993 (49)			•																			
Brinton et al, 1986(70)													•									
Buckley et al, 2015 (42)	•				•																	
Byrne et al, 1991(50)			•																			
Byrne et al, 2000 (71)													•									
Byrne et al, 2001 (15)													•		•							
Castells et al, 2015 (75)			•																			
Collins et al, 2006 (16)			•																			
Collins et al, 2007 (54)					•							•										
Cote et al, 2012 (86)	•			•																		
Dupont et al, 1985 (17)	•		•											•								
Dupont et al, 1987 (58)											•											
Dupont et al, 1989(47)			•			•	•	•	•				•			•						
Dupont et al, 1993 (65)			•									•		•								
Dupont et al, 1994 (53)			•																			
Dupont et al, 1999 (68)													•									
Figueroa et al, 2021 (62)			•								•	•					•					
Gail et al, 2007 (90)																		•				
Gallicchio et al, 2006 (46)		•	•			•	•	•		•	•								•			
Ghosh et al, 2010 (60)		•								•		•	•		•							
Hartmann et al, 2005 (18)	•		•									•										
Hartmann et al, 2014 (27)	•		•											•								
Horn-Ross et al, 2004 (81)						•																
Hutchinson et al, 1980 (39)	•													•								
Jensen et al, 1989 (51)			•																			
Kabat et al, 2010a (43)	•		•		•							•										
Kabat et al, 2010b (61)		•	•				•		•		•	•	•								•	
Kilgore et al, 2021 (82)	•			•																•		
Krieger et al, 1992 (40)	•				•																	
Lilleborge et al, 2021 (66)		•				•	•						•									•
Lubin et al, 1983 (38)	•				•																	
Magnusson et al, 1998 (85)			•																			
Marshall et al, 1997 (55)					•																	
McCredie et al, 1997 (52)			•																			
McDivitt et al, 1992 (87)			•																			
Meares et al, 2016 (29)	•																					
Milanese et al, 2006 (59)											•											
Moon et al, 2014 (44)	•																					
Nassar et al, 2015 (30)	•		•																			
Newcomb et al, 1995 (69)													•									
Page et al, 1985 (31)	•		•																			
Page et al, 2003 (32)	•											•										
Pankratz et al, 2008 (9)	•		•					•			•							•				
Posso et al, 2022 (72)														•								
Román et al, 2021 (19)															•							
Román et al, 2022 (45)	•				•																	
Said et al, 2015(33)	•		•																			
Schonberg et al, 2016 (91)																		•				
Sherman et al, 2020 (92)																			•			
Stanford et al, 1995 (67)													•									
Tamimi et al, 2005 (88)						•																
Tamimi et al, 2010 (56)								•								•						
Thomas et al, 1982 (63)								•			•	•				•	•					
Tice et al, 2013 (21)															•							
Tice et al, 2015(6)	•																					
Vierkant et al, 2017 (22)															•							
Visscher et al, 2014 (34)	•		•																			
Whiffen et al, 2011 (64)		•	•			•	•		•		•											
Worsham et al, 2007 (37)	•																					
Worsham et al, 2009 (48)		•	•	•			•		•	•		•	•				•					
Yaghjyan et al, 2015 (74)															•							
Yoon et al, 2020 (36)	•													•	•							
CDC CSHS, 1983 (89)									•													

Supplementary Table 5: Joanna Briggs Institute Critical Appraisal Checklist for Cohort Studies

JBI CRITICAL APPRAISAL CHECKLIST FOR COHORT STUDIES

Reviewer: Date:

Author: Year:

	Yes	No	Unclear	Not applicable
1. Were the two groups similar and recruited from the same population?	☐	☐	☐	☐
2. Were the exposures measured similarly to assign people to both exposed and unexposed groups?	☐	☐	☐	☐
3. Was the exposure measured in a valid and reliable way?	☐	☐	☐	☐
4. Were confounding factors identified?	☐	☐	☐	☐
5. Were strategies to deal with confounding factors stated?	☐	☐	☐	☐
6. Were the groups/participants free of the outcome at the start of the study (or at the moment of exposure)?	☐	☐	☐	☐
7. Were the outcomes measured in a valid and reliable way?	☐	☐	☐	☐
8. Was the follow up time reported and sufficient to be long enough for outcomes to occur?	☐	☐	☐	☐
9. Was follow up complete, and if not, were the reasons to loss to follow up described and explored?	☐	☐	☐	☐
10. Were strategies to address incomplete follow up utilized?	☐	☐	☐	☐
11. Was appropriate statistical analysis used?	☐	☐	☐	☐

Overall appraisal: Include ☐ Exclude ☐ Seek further info ☐

Comments (Including reason for exclusion):

Supplementary Table 6: Joanna Briggs Institute Critical Appraisal Checklist for Case-Control Studies

JBI CRITICAL APPRAISAL CHECKLIST FOR CASE CONTROL STUDIES

Reviewer: Date:

Author: Year:

	Yes	No	Unclear	Not applicable
1. Were the groups comparable other than the presence of disease in cases or the absence of disease in controls?	☐	☐	☐	☐
2. Were cases and controls matched appropriately?	☐	☐	☐	☐
3. Were the same criteria used for identification of cases and controls?	☐	☐	☐	☐
4. Was exposure measured in a standard, valid and reliable way?	☐	☐	☐	☐
5. Was exposure measured in the same way for cases and controls?	☐	☐	☐	☐
6. Were confounding factors identified?	☐	☐	☐	☐
7. Were strategies to deal with confounding factors stated?	☐	☐	☐	☐
8. Were outcomes assessed in a standard, valid and reliable way for cases and controls?	☐	☐	☐	☐
9. Was the exposure period of interest long enough to be meaningful?	☐	☐	☐	☐
10. Was appropriate statistical analysis used?	☐	☐	☐	☐

Overall appraisal: Include ☐ Exclude ☐ Seek further info ☐

Comments (Including reason for exclusion):

Supplementary Table 7: Quality appraisal and risk of bias assessment (n=67 studies).

	**First author, year**
**Cohort studies**	
Low risk of bias (≤1 unmet criteria on checklist)	Buckley *et al*, 2015 (42); Castells *et al*, 2015 (75); Cote *et al,* 2012 (86); Dupont *et al,* 1985 (17); Dupont *et al,* 1987 (58); Dupont *et al,* 1989 (47); Dupont *et al,* 1994 (53); Gallicchio *et al,* 2006 (46); Ghosh *et al,* 2010 (60); Horn-Ross *et al,* 2004 (81); Lilleborge *et al,* 2021 (66); Page *et al,* 1985 (31); Page *et al,* 2003 (32); Pankratz *et al,* 2008 (9); Posso *et al,* 2022 (72); Román *et al,* 2021(19) ; Román *et al,* 2022 (45); Schonberg *et al,* 2016 (90); Sherman *et al,* 2020 (91); Thomas *et al,* 1982 (63); Tice *et al,* 2013 (21); Tice *et al,* 2015 (6)
Moderate risk of bias (2-3 unmet criteria on checklist)	Ashbeck *et al,* 2007 (41); Bodian *et al,* 1993 (49); Dupont *et al,* 1999 (68); Hartmann *et al,* 2005 (18); Hartmann *et al,* 2014 (27); Hutchinson *et al,* 1980 (39); Jensen *et al,* 1989 (51); Kilgore *et al,* 2021 (82); Krieger *et al,* 1992 (40); Meares *et al,* 2016 (29); Milanese 2006 (59); Moon *et al,* 2014 (44); Nassar *et al,* 2015 (30); Said *et al,* 2015 (33); Tamimi *et al,* 2010 (56); Vierkant *et al,* 2017 (22); Visscher *et al,* 2014 (34); Worsham *et al,* 2007 (37)
High risk of bias (>3 unmet criteria on checklist)	Whiffen *et al,* 2011 (64); Yoon *et al,* 2020 (36)
**Case-control studies**	
Low risk of bias (≤1 unmet criteria on checklist)	Abubakar *et al,* 2021 (73); Aroner *et al,* 2013 (28); Arthur *et al,* 2017 (57); Brinton *et al,* 1986 (70); Byrne *et al,* 1991 (50); Byrne *et al,* 2000 (71); Byrne *et al,* 2001 (15); CDC Cancer and Steroid Hormone Study, 1983 (89); Collins *et al,* 2006 (16); Collins *et al,* 2007 (54); Figueroa *et al,* 2021 (62); Gail *et al,* 2007 (92); Kabat *et al,* 2010a (43); Kabat *et al,* 2010b (61); Lubin *et al,* 1983 (38); Marshall *et al,* 1997 (55); Stanford *et al,* 1995 (67); Tamimi *et al,* 2005 (88); Worsham *et al,* 2009 (48); Yaghjyan *et al,* 2015 (74)
Moderate risk of bias (2-3 unmet criteria on checklist)	Dupont *et al,* 1993 (65); Magnusson *et al,* 1998 (85); McCredie *et al,* 1997 (52); McDivitt *et al,* 1992 (87); Newcomb *et al,* 1995 (69)
High risk of bias (>3 unmet criteria on checklist)	*(none)*
